# Financial burden and impact of atopic dermatitis out-of-pocket healthcare expenses among black individuals in the United States

**DOI:** 10.1007/s00403-021-02282-3

**Published:** 2021-09-27

**Authors:** Raj Chovatiya, Wendy Smith Begolka, Isabelle J. Thibau, Jonathan I. Silverberg

**Affiliations:** 1 Department of Dermatology, Northwestern University Feinberg School of Medicine, Chicago, IL 60611 USA; 2 National Eczema Association, 505 San Marin Dr #B300, Novato, CA 94945 USA; 3 Department of Dermatology, The George Washington University School of Medicine and Health Sciences, Washington, DC 20037 USA

**Keywords:** Atopic dermatitis, Out-of-pocket, Expense, Cost of care, Financial burden

## Abstract

Black race is associated with increased atopic dermatitis (AD) severity and healthcare resource utilization. However, the burden of out-of-pocket (OOP) expenses among black individuals with AD is not well understood. We sought to characterize the categories and impact of OOP healthcare expenses associated with AD management among black individuals. A 25-question voluntary online survey was administered to National Eczema Association members (*N* = 113,502). Inclusion criteria (US residents age ≥ 18 years; self-report of AD or primary caregivers of individuals with AD) was met by 77.3% (1118/1447) of respondents. Black individuals with AD were younger, had lower household income, Medicaid, urban residence, poor AD control and frequent skin infections (*P* ≤ 0.02). Blacks vs. non-blacks reported more OOP costs for prescription medications covered (74.2% vs. 63.6%, *P* = 0.04) and not covered (65.1% vs. 46.5%, *P* = 0.0004) by insurance, emergency room visits (22.1% vs. 11.8%, *P* = 0.005), and outpatient laboratory testing (33.3% vs. 21.8%, *P* = 0.01). Black race was associated with increased household financial impact from OOP expenses (*P* = 0.0009), and predictors of financial impact included minimally controlled AD (adjusted OR [95% CI] 13.88 [1.63–117.96], *P* = 0.02), systemic therapy (4.34 [1.63–11.54], 0.003), > $200 monthly OOP expenses (14.28 [3.42–59.60], *P* = 0.0003), and Medicaid (4.02 [1.15–14.07], *P* = 0.03). Blacks with Medicaid had higher odds of harmful financial impact (3.32 [1.77–6.24], *P* = 0.0002) than those of black race (1.81 [1.04–3.15], *P* = 0.04) or with Medicaid (1.39 [1.02–1.88], *P* = 0.04) alone. Black race is associated with increased OOP costs for AD and significant household financial impact. Targeted interventions are needed to address financial disparities in AD.

**Supplementary Information:**

The online version contains supplementary material available at 10.1007/s00403-021-02282-3.

## Introduction

Atopic dermatitis (AD) is one of the most common chronic inflammatory skin diseases among both United States (US) children and adults. Previous studies found the US prevalence of AD to be 7% in adults and 13% in children [1–3] AD is characterized by heterogenous skin lesions with varying distribution [4], a constellation of symptoms including itch, skin-pain [5], and mental health disturbances [6], numerous atopic and non-atopic comorbid health conditions [7, 8], and complex course consisting of variable persistence, flares, and severity [9, 10]. Significant morbidity associated with AD contributes to reduced health-related quality of life (HRQoL) and immense psychosocial burden [1, 11, 12].

Clinical phenotype and patient-burden of AD vary across racial and ethnic groups [13]. This heterogeneity is likely driven by a complex interplay of intrinsic (e.g., genetics, skin barrier dysfunction, cutaneous immune skewing, comorbidities) and extrinsic (e.g., socioeconomic status, geographic location, environment/climate) factors [13–15]. Black individuals have a higher prevalence of AD [16–18], higher burden of moderate-to-severe disease [19], increased rates of allergic comorbidities [20], greater AD-related impact on HRQoL [21], and often present with more treatment-resistant disease [15]. These features can make long-term AD control quite difficult. Given variable long-term efficacy and safety of many current treatments, healthcare providers (HCPs) and patients frequently have to combine therapies, seek new treatments, and consider adjunctive approaches to achieve optimal results—all of which contribute increased costs.

Atopic dermatitis (AD) is associated with a considerable financial burden, consisting of elevated direct costs related to healthcare resource utilization and indirect societal costs secondary to lost work productivity [22–27]. From the perspective of individual households, out-of-pocket (OOP) expenses are an essential and tangible element in routine management of finances. Previous population-based studies showed multifactorial increases in overall OOP expenses in AD patients [28, 29], and more recently, it was shown that individuals with AD report a wide variety of distinct medical and non-medical OOP healthcare costs related to AD care [30]. While black race was shown to be associated with increased healthcare resource utilization in AD [26], little is known about OOP healthcare expenses related to management of AD care among black individuals. We hypothesized that black race is associated with unique categories of OOP healthcare expenses and increased household financial impact. In this study, we sought to characterize OOP costs and financial impact in black individuals with AD.

## Methods

### Study design

A 25-question voluntary survey was administered online to all National Eczema Association (NEA) members (> 110,000 individuals with AD and family members) between November–December 2019. Electronic informed consent was obtained, and respondents completing the survey were given the option to enter a drawing to win one of ten $50 gift cards. Survey response was not linked to gift card drawing or distribution. Inclusion criteria included US residency, age ≥ 18 years, and either self-report or primary caregiver of children or young adult with AD. The survey was started by 1447 persons, and 1118 (77.3%) met inclusion criteria.

### Survey questions

Diagnosis of AD was determined by yes/no response to “Have [you/the person with atopic dermatitis] been diagnosed with atopic dermatitis by a healthcare provider?” Demographics were collected, including age, gender, race/ethnicity, household income, insurance status, and geographical setting. Current AD severity (clear/mild/moderate/severe), control (very well/moderately well/somewhat/minimally/not controlled), number of flare days in the last month (0/1–3/4–7/8–10/ ≥ 11), number of HCP visits in the past year (0/1/2/3/4/5/ ≥ 6), HCP-diagnosed comorbid health conditions (asthma/allergic rhinitis/food allergy/frequent and/or persistent skin infections/anxiety and/or depression), total number of prescriptions in the past year (0/1/2/3/4/5/ ≥ 6, and specific topical (antimicrobials/corticosteroids/crisaborole /tacrolimus/pimecrolimus) and systemic (phototherapy/dupilumab/azathioprine/cyclosporine /methotrexate /mycophenolate mofetil/tacrolimus/oral corticosteroids/injectable corticosteroids) prescription therapies were also queried.

Respondents were also asked about: OOP expenses related to evaluation or treatment of AD in the past 30 days for current medical approaches; total yearly OOP expenses for AD; and impact of yearly OOP expenses on household finances (none/minimal/moderate/significant/devastating).

### Data analysis

Statistical analysis was performed using SAS version 9.4 (SAS Institute, Cary, NC). Chi-squared tests were used for comparisons of categorical variables including sociodemographics factors, AD severity and control measures, and categories of OOP expenses. Kruskal–Wallis one-way analysis of variance was used for comparison of median annual OOP costs. Predictors of financial impact were determined by multivariable logistic regression with invoked backward elimination stepwise selection with financial impact as the dependent variable. Two-way interactions of race and insurance on financial impact were assessed using bivariable and multivariable logic regression and included in models if significant (*P* < 0.05) and they modified effect size by ≥ 20%. Corrected *P* values ≤ 0.05 were considered significant.

## Results

### Respondent characteristics and disease burden

Overall, respondents included adults with AD (% prevalence [frequency]: 77.5% [866]) and parents and/or primary caregivers of children, teens, or young adults with AD (22.5% [252]). Most respondents were white (72.4% [697]), followed by black/African-American (10.6% [102]), multiracial (6.5% [63]), Asian (6.0% [58]), other (2.9% [28]), American Indian/Alaskan Native (0.8% [8]), and Native Hawaiian/Pacific-Islander (0.7% [7]). Black vs. non-black individuals with AD were more likely to be younger (≤ 35 years: 55.9% vs. 42.7%, *P* = 0.001), non-Hispanic (97.1% vs. 89.7%, *P* = 0.02), have lower household income (≤ $24,999: 31.7% vs. 16.8%, *P* = 0.005), Medicaid or state assistance (20.8% vs. 8.4%, *P* = 0.0002), and live in an urban setting (41.2% vs. 21.8%, *P* < 0.0001) (Table S1). Black vs. non-black respondents also had poorer disease control (minimally or somewhat controlled: 63.8% vs. 50.3%, *P* = 0.02), increased rates of frequent/persistent skin infections (28.4% vs. 18.1%, *P* = 0.01), and lower rates of anxiety and/or depression (24.5% vs. 38.0%, *P* = 0.008).

### OOP expenses

Black vs. non-black respondents were more likely to report OOP expenses for prescription medication co-pays covered by insurance (74.2% vs. 63.6%, *P* = 0.04), emergency room visits (22.1% vs. 11.8%, *P* = 0.005), prescription medications not covered by insurance (65.1% vs. 46.5%, *P* = 0.0004), and outpatient laboratory testing (33.3% vs. 21.8%, *P* = 0.01) (Table 1). Numerically higher proportions of black respondents also reported OOP expenses for co-pays and/or deductibles for doctor or other HCP office visits, hospitalization, anti-itch medications, pain medications, sleep medications, hygiene products, childcare, and transportation.

**Table 1:** Categories of OOP expenses stratified by black race

Variable	Overall (*n* = 1018)	Black race		
		No (*n* = 861)	Yes (*n* = 102)	*P* value
Healthcare providers and prescriptions				
Deductible	686 (68.7%)	575 (68.3%)	70 (70.7%)	0.62
Hospitalization	23 (2.5%)	17 (2.2%)	4 (4.3%)	0.22
Prescriptions covered	635 (64.3%)	530 (63.6%)	72 (74.2%)	0.04
Emergency room visits	123 (13.3%)	92 (11.8%)	21 (22.1%)	0.005
Prescriptions not covered	468 (48.6%)	377 (46.5%)	64 (65.1%)	0.0004
Lab testing	216 (23.2%)	171 (21.8%)	31 (33.3%)	0.01
Outpatient phototherapy	42 (4.6%)	34 (4.4%)	4 (4.4%)	0.99
Mental health services	133 (14.4%)	111 (14.3%)	12 (12.8%)	0.68
Non-prescription health products				
Moisturizers	934 (94.3%)	800 (94.2%)	94 (94.0%)	0.93
Anti-itch meds	647 (68.3%)	542 (66.8%)	73 (75.3%)	0.09
Allergy meds	715 (75.1%)	609 (75.0%)	70 (70.7%)	0.35
Pain meds	449 (49.3%)	376 (48.4%)	49 (51.6%)	0.56
Sleep meds	336 (37.0%)	283 (36.4%)	37 (39.8%)	0.52
Bandages	446 (48.4%)	400 (50.8%)	27 (28.4%)	< 0.0001
Hygiene products	824 (85.0%)	703 (84.6%)	89 (89.0%)	0.24
Supplements	491 (52.2%)	428 (53.2%)	43 (44.3%)	0.10
Complementary approaches and care coordination				
Alternative therapy	180 (19.0%)	157 (19.2%)	18 (18.2%)	0.80
Childcare	48 (5.2%)	40 (5.2%)	6 (6.5%)	0.60
Adjunctive therapy	150 (15.9%)	135 (16.6%)	10 (10.2%)	0.10
Specialized cleaning products	732 (74.7%)	635 (75.1%)	74 (73.3%)	0.69
Specialized clothing and bedding	430 (44.8%)	372 (45.0%)	42 (42.4%)	0.63
Transportation	444 (46.8%)	370 (46.4%)	52 (54.2%)	0.15

**Fig. 1:**
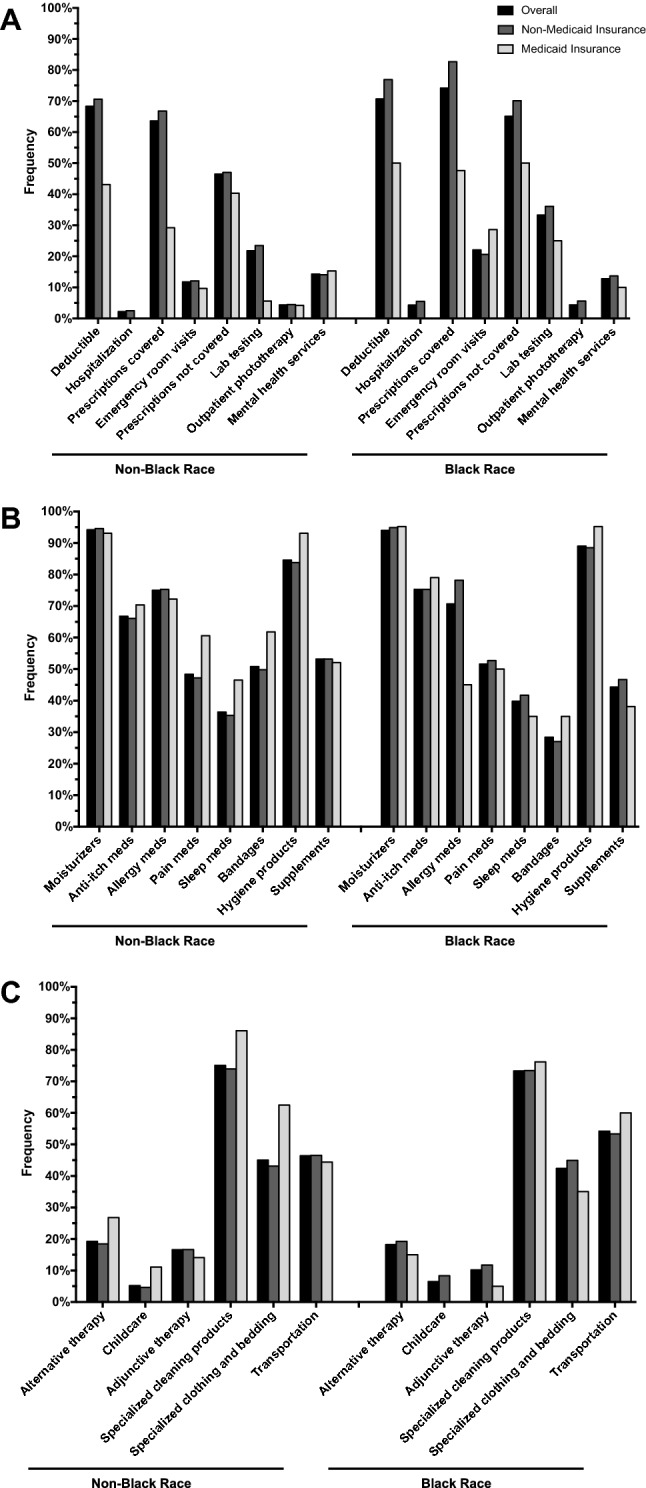
Categories of OOP expense stratified by race and insurance. **A** OOP expenses for healthcare providers and prescriptions, **B** OOP expenses for non-prescription health products, **C** OOP expenses for complementary approaches and care coordination

Given the significant proportion of black respondents reporting use of Medicaid, OOP costs were further stratified by insurance status. Fewer black individuals with vs. without Medicaid insurance reported OOP costs for deductibles for HCP office visits (50.0% vs. 76.9%), hospitalization (0% vs. 5.5%), prescription medication co-pays covered by insurance (47.6% vs. 82.7%), prescription medications not covered by insurance (50.0% vs. 70.1%), and lab testing (25.0% vs. 36.1%) (Fig. 1A). Similar findings were seen in a number of other categories of non-prescription health products and complementary approaches and care coordination including (Fig. 1B, C).

**Fig. 2:**
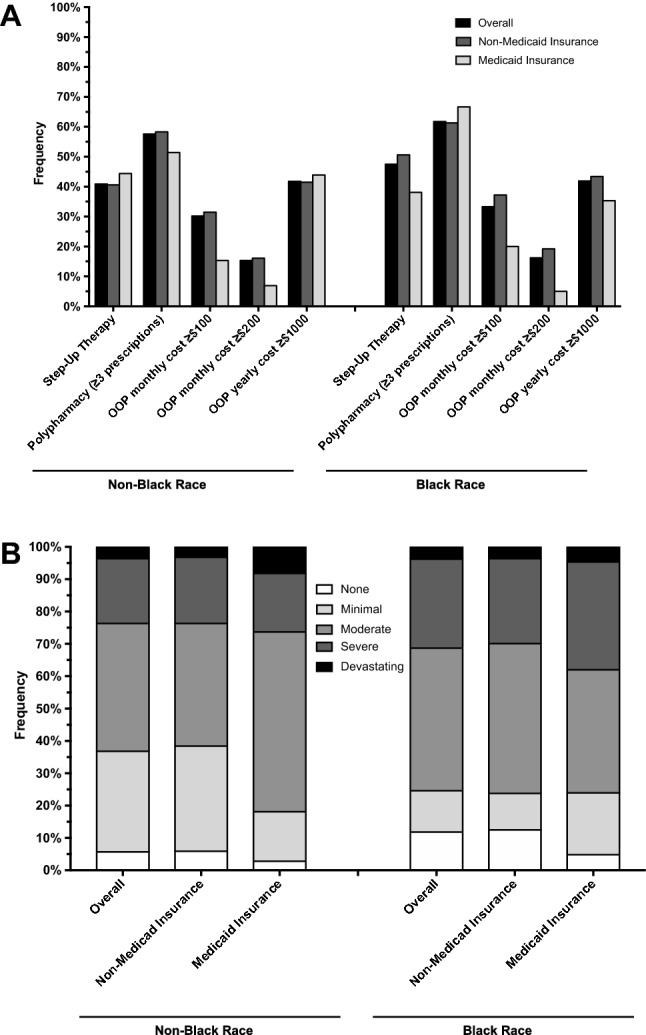
Associations and impacts of OOP expenses stratified by race and insurance. **A** Prescription therapies and total OOP costs, **B** household financial impact

A numerically higher proportion of black respondents reported use of step-up therapy (i.e., systemic therapy including injectable, oral, or phototherapy), prescription polypharmacy (≥ 3 prescriptions), and higher monthly OOP costs for co-pays and/or deductibles for HCP office visits (Table 2). In contrast, respondents with Medicaid insurance reported significantly lower monthly OOP expenses in the same category and a numerically lower median OOP yearly cost. Black respondents with vs. without Medicaid insurance had a lower frequency of step-up therapy, higher rate of polypharmacy, and lower OOP monthly and yearly costs (Fig. 2A).

**Table 2:** Associations and impact of OOP expenses by race or insurance

Variable	Overall (*n* = 1018)	Black race			Medicaid insurance		
		No (*n* = 861)	Yes (*n* = 102)	*P* value	No (*n* = 861)	Yes (*n* = 93)	*P* value
Treatments							
Step-up therapy	442 (41.0%)	350 (40.9%)	48 (47.5%)	0.20	355 (41.5%)	40 (43.0%)	0.78
Polypharmacy (≥ 3 prescriptions)	617 (57.5%)	496 (57.6%)	63 (61.8%)	0.67	504 (58.5%)	51 (54.8%)	0.78
OOP costs for co-pays/deductibles for HCP visits in past 30 days—freq (%)							
≥ $100	311 (31.1%)	254 (30.2%)	33 (33.3%)	0.51	269 (32.0%)	15 (16.3%)	0.002
≥ $200	158 (15.8%)	129 (15.3%)	16 (16.2%)	0.83	138 (16.4%)	6 (6.5%)	0.01
OOP yearly cost—freq (%)							
≥ $1000	364 (41.9%)	323 (41.8%)	39 (41.9%)		324 (41.7%)	35 (42.2%)	0.93
OOP yearly cost—median (min, max)	600 (0, 200,000)	550 (0, 200,000)	700 (10, 16,000)	0.64	600 (0, 200,000)	500 (12, 10,000)	0.32
Household financial impact—freq (%)							
None	61 (6.3%)	49 (5.7%)	12 (11.8%)	0.0009	56 (6.5%)	3 (3.2%)	0.005
Minimal	281 (29.1%)	267 (31.1%)	13 (12.8%)		262 (30.5%)	15 (16.1%)	
Moderate	387 (40.1%)	339 (39.5%)	45 (44.1%)		332 (38.7%)	48 (51.6%)	
Severe	201 (20.8%)	173 (20.1%)	28 (27.5%)		181 (21.1%)	20 (21.5%)	
Devastating	36 (3.7%)	31 (3.6%)	4 (3.9%)		28 (3.3%)	7 (7.5%)

### Impact of OOP expenses

Black race was associated with more harmful impact of OOP expenses for AD on personal/family finances (*P* = 0.0009) (Table 2). More black vs. non-black individuals with AD reported moderate (44.1% vs. 39.5%), severe (27.5% vs. 20.1%), and devastating (3.9% vs. 3.6%) household financial impact. Medicaid vs. non-Medicaid insurance was similarly associated with a higher degree of moderate (51.6% vs. 38.7%), severe (21.5% vs. 21.1%), and devastating (7.5% vs. 3.3%) household financial impact (*P* = 0.005). A numerically higher proportion of black respondents with vs. without Medicaid insurance reported severe or devastating financial impact (Fig. 2B).

Positive predictors of harmful financial impact among blacks with AD included minimally controlled AD (adjusted odds ratio [95% confidence interval], P value: 13.88 [1.63–117.96], *P* = 0.02), comorbid anxiety and/or depression (4.34 [1.37–13.70], *P* = 0.01), step-up therapy (4.34 [1.63–11.54], 0.003), > $200 spent on monthly OOP expenses for co-pays and/or deductibles for HCP office visits (14.28 [3.42–59.60], *P* = 0.0003), and Medicaid insurance (4.02 [1.15–14.07], *P* = 0.03) (Table 3). Significant two-way interactions occurred for black race with Medicaid insurance as predictors of harmful financial impact (Table 4). That is, individuals of black race with Medicaid insurance had higher odds of harmful impact of AD OOP expenses on household finances (3.32 [1.77–6.24], *P* = 0.0002) than those of black race (1.81 [1.04–3.15], *P* = 0.04) or with Medicaid insurance alone (1.39 [1.02–1.88], *P* = 0.04).

**Table 3:** Predictors of financial impact by race

Non-black race			Black race		
Variables	Adjusted odds ratio	*P* value	Variables	Adjusted odds ratio	*P* value
Household income ($)			Age (yr)		
≤ 24,999	1.00 [ref]	–	≤ 2	1.00 [ref]	–
25,000–99,999	0.62 [0.42–0.92]	0.02	3–5	4.88 [0.27–87.88]	0.28
≥ 100,000	0.24 [0.16–0.37]	< 0.0001	6–11	2.74 [0.34–22.01]	0.34
Current AD severity			12–17	5.87 [0.47–74.02]	0.17
Clear	1.00 [ref]	–	18–25	0.22 [0.03–1.53]	0.13
Mild	0.96 [0.39–2.34]	0.93	26–35	0.27 [0.03–2.53]	0.25
Moderate	1.59 [0.67–3.77]	0.30	36–50	0.48 [0.07–3.52]	0.47
Severe	3.02 [1.24–7.36]	0.02	51–64	2.52 [0.348–18.29]	0.36
Asthma			≥ 65	0.05 [0.01–0.46]	0.01
No	1.00 [ref]	–	Current AD control		
Yes	1.41 [1.05–1.89]	0.02	Very well controlled	1.00 [ref]	–
HCP visits in past year			Moderately well controlled	3.18 [0.43–23.71]	0.26
0	1.00 [ref]	–	Somewhat controlled	2.50 [0.38–16.35]	0.34
1–2	1.40 [0.84–2.33]	0.20	Minimally controlled	13.88 [1.63–117.96]	0.02
3–4	1.68 [0.97–2.91]	0.06	Anxiety and/or depression		
≥ 5	2.94 [1.64–5.26]	0.0003	No	1.00 [ref]	–
OOP co-pays and/or deductibles for healthcare provider office visits in past 30 days			Yes	4.34 [1.37–13.70]	0.01
≤ $200	1.00 [ref]	–	Step-up therapy		
> $200	1.95 [1.28–2.99]	0.002	No	1.00 [ref]	–
Annual OOP expenses			Yes	4.34 [1.63–11.54]	0.003
≤ $1000	1.00 [ref]	–	Medicaid insurance		
> $1000	4.87 [3.46–6.86]	< 0.0001	No	1.00 [ref]	–
			Yes	4.02 [1.15–14.07]	0.03
			OOP co-pays and/or deductibles for healthcare provider office visits in past 30 days		
			≤ $200	1.00 [ref]	–
			> $200	14.28 [3.42–59.60]	0.0003

**Table 4:** Effect of interaction between black race and medicaid insurance as predictors of household financial impact due to OOP expenses

Black race	Medicaid insurance	Crude OR [95% CI]	*P* value	Adjusted OR [95% CI]	*P* value
No	No	1.00 [ref]		1.00 [ref]	
Yes	No	1.74 [1.04–2.93]	0.04	1.81 [1.04–3.15]	0.04
No	Yes	1.34 [1.01–1.79]	0.04	1.39 [1.02–1.88]	0.04
Yes	Yes	2.11 [1.17–3.82]	0.01	3.32 [1.77–6.24]	0.0002

Adjusted model includes gender, geographic settings, measures of disease activity (severity, control, flares), number of healthcare provider visits, total number of prescription medications, step-up therapy, comorbidities (allergic rhinitis, asthma, skin infections, anxiety and/or depression), and monthly OOP expenses as covariates

## Discussion

In this study, we found that black respondents with AD were significantly more likely to report OOP costs for prescription medications both covered and not covered by insurance, emergency room visits, and outpatient laboratory testing. More black individuals also reported OOP costs for office visit co-pays/deductibles, a variety of OTC medications, hygiene products, childcare, and transportation. Despite elevated OOP costs across a variety of AD healthcare categories, black respondents were more likely to have a lower household income than their non-black counterparts, and they were also more likely to report a severe or devastating financial impact on household finances. Black race itself was found to be a predictor of harmful financial impact among individuals with AD. Taken together, these findings underscore the real-world OOP expense burden faced by black Americans with AD.

In the surveyed population, black individuals with AD were significantly more likely to be younger, live in an urban setting, use Medicaid insurance and have poorer disease control. In addition, predictors of harmful financial impact due to OOP expenses among blacks with AD included minimally controlled AD, systemic therapy, Medicaid insurance, and increased OOP expenses for HCP co-pays and/or deductibles. Previous studies showed that AD is more prevalent among black children in the US [18, 31, 32], and they are nearly twofold more likely to develop AD than their white counterparts, even after adjusting for sociodemographic factors [31]. Urbanization is also associated with increased risk of AD [33, 34], with disease severity driven in part by differences in environmental factors (e.g., hygiene, pollution, exposure to infectious disease) and their interaction with skin of different races [35]. While black individuals are less likely to pursue dermatologic care overall, they are nearly threefold more likely to be diagnosed with AD during an office visit [36], and they are also more likely to have moderate-to-severe AD [19]. Those that do end up seeking outpatient AD care have an increased number of visits and high number of prescription medications compared to whites [37]. In a U.S population-based study, individuals with AD more frequently reported not being able to afford prescription medications and receive timely care [28]. Black Americans in general are more likely to be underinsured and have difficulty in obtaining medical care [38]. For those with Medicaid insurance, there are limited options for dermatologic care. A survey of dermatologists conducted by the American Academy of Dermatology reported that only 5% of practices accepted patients with Medicaid, far less than would be predicted based on the percentage of the US population receiving Medicaid at the time of the study [39]. Our findings reflect these racial and socioeconomic disparities, provide evidence for increased financial burden among blacks with AD, and support the need for targeted strategies to address these inequities.

US adults with AD have high rates of emergency department (ED) and urgent care visits, and these are more common among blacks, those with lower household income, and those with prescriptions not covered by their insurance provider [26]. Frequency and costs of ED visits related to AD have risen over the past decade [23]. Regular use of ED care for chronic disease management is a major strain on individual and global healthcare finances and is severalfold more expensive than an outpatient office visit [40]. Black race, along with public insurance and lower household income, has also been showed to be associated with increased primary hospitalization for AD [24]. This pattern of care utilization among blacks with AD, consisting of fewer outpatient office visits, increased prescription medications, increased ED visits, and higher risk of hospitalization, in conjunction with our findings of significantly increased OOP expenditures in ED, medication, and laboratory testing categories, reflects immense individual efforts to manage a high burden of disease. While no single intervention will lower OOP costs and improve access to AD care, a multifaceted strategy to optimize outpatient care could include: better training of primary care HCPs to recognize and treat mild-to-moderate AD; a streamlined referral system with faster access to specialists such as dermatologists, especially for AD flares and management of any comorbid conditions; more broadly inclusive insurance coverage; and expanded use of teledermatology to better reach those who are unable to see a dermatologist.

While OOP costs were increased in several distinct categories and there was a higher proportion of Medicaid insurance use among blacks with AD, further stratification of black race by Medicaid insurance did not reveal any significant areas of expense. Several OOP cost categories decreased among blacks with vs. without Medicaid. This likely stems from the structure of Medicaid itself, which has stringent guidelines for cost-sharing and limits OOP cost to no more than 5% of household income. Despite placing strict limits on OOP expenses and increasing overall access to care, we found that Medicaid insurance was still an independent predictor of financial impact due to OOP expenses, highlighting the financial difficulties AD patients continue to face due to limitations of Medicaid coverage. More so, black race and Medicaid insurance exhibited a two-way interaction and was associated with an even higher risk of harmful financial impact, higher than that due to either factor alone. HCPs should recognize the immense financial burden in this group of patients and proactively discuss financial impact of OOP costs alongside efficacy and safety when counseling patients. There is no “ideal” or “one-size-fits-all” treatment plan for AD. Rather, HCPs should engage in shared decision making with their AD patients—especially black patients—and create an individualized treatment plan that is practical, feasible, and financially responsible.

Study strengths include a large, racially diverse cohort of AD patients and caregivers with assessment of AD severity, control, expenses, and financial impact. The inclusion of 22 unique categories of OOP expenses allowed for accurate understanding of financial burden. The cross-sectional design of this study is an important limitation as we were unable to assess longitudinal changes in cost and impact. Though selection bias is possible given that this was an internet-based survey to the NEA membership, the respondent demographics were well distributed across races, geographic location, insurance, income, and AD severity. While self-report of costs may not be as accurate as claims analysis, direct response from patients and caregivers allows for more complete assessment of disease state and household finances. Additional studies are needed to confirm these findings and better understand OOP expenses across other races and socioeconomic groups.

In conclusion, among individuals with AD, black race is associated with increased OOP expenses in a variety of unique healthcare categories and significant household financial impact. Additional studies are needed to better understand unique OOP financial considerations among black individuals and develop targeted approaches to reduce both the financial and overall burden of AD.

## Supplementary Information

Below is the link to the electronic supplementary material.

Supplementary file1 (DOCX 29 kb)

## Abbreviations

AD: Atopic dermatitis
OOP: Out-of-pocket
OTC: Over the counter
HRQoL: Health-related quality of life
HCP: Healthcare provider
