# Supplementary material for: Financial burden and impact of atopic dermatitis out-of-pocket healthcare expenses among black individuals in the United States
Source: Arch Dermatol Res. 2021 Sep 27;314(8):739–47. doi: 10.1007/s00403-021-02282-3 (PMC9399197; doi:10.1007/s00403-021-02282-3)
Supplement: Supplementary file 1 — Supplementary file1 (DOCX 29 kb) [file 403_2021_2282_MOESM1_ESM.docx]

| **Supplemental Table 1: Respondent characteristics and disease burden** | | | | |
| --- | --- | --- | --- | --- |
|  | **Overall (n=1,018)** | **Black Race** | | |
| **Variable – freq (%)** |  | **No**  **(n=861)** | **Yes**  **(n=102)** | **P-value** |
| Eczema relationship |  |  |  |  |
| Caregiver (<18 years) | 224 (20.0%) | 169 (19.6%) | 30 (29.4%) | 0.05 |
| Caregiver (18-25 years) | 28 (2.5%) | 23 (2.7%) | 1 (1.0%) |  |
| Patient (≥18 years) | 866 (77.5%) | 669 (77.7%) | 71 (69.5%) |  |
| Age (yr) |  |  |  |  |
| ≤2 | 42 (3.8%) | 28 (3.3%) | 9 (8.8%) | 0.001 |
| 3-5 | 69 (6.2%) | 59 (6.9%) | 3 (2.9%) |  |
| 6-11 | 68 (6.1%) | 50 (5.8%) | 12 (11.8%) |  |
| 12-17 | 49 (4.4%) | 36 (4.2%) | 6 (5.9%) |  |
| 18-25 | 139 (12.4%) | 98 (11.4%) | 18 (17.7%) |  |
| 26-35 | 130 (11.6%) | 97 (11.3%) | 9 (8.8%) |  |
| 36-50 | 173 (15.5%) | 127 (14.8%) | 19 (18.6%) |  |
| 51-64 | 247 (22.1%) | 203 (23.6%) | 15 (14.7%) |  |
| ≥65 | 201 (18.0%) | 163 (18.9%) | 11 (10.8%) |  |
| Gender |  |  |  |  |
| Female | 855 (76.5%) | 656 (76.2%) | 82 (80.4%) | 0.6 |
| Male | 251 (22.5%) | 195 (22.7%) | 20 (19.6%) |  |
| Non-binary/other | 4 (0.4%) | 4 (0.5%) | 0 (0.0%) |  |
| Prefer not to answer | 8 (0.7%) | 6 (0.7%) | 0 (0.0%) |  |
| Hispanic ethnicity |  |  |  |  |
| No | 871 (90.5%) | 772 (89.7%) | 99 (97.1%) | 0.02 |
| Yes | 92 (9.6%) | 89 (9.2%) | 3 (2.9%) |  |
| Household income ($) |  |  |  |  |
| ≤24,999 | 175 (18.3%) | 143 (16.8%) | 32 (31.7%) | 0.005 |
| 25,000-49,999 | 190 (19.9%) | 177 (20.8%) | 13 (12.9%) |  |
| 50,000-74,999 | 192 (20.1%) | 172 (20.2%) | 20 (19.8%) |  |
| 75,000-99,999 | 122 (12.8%) | 110 (12.9%) | 12 (11.9%) |  |
| 100,000-124,999 | 103 (10.8%) | 90 (10.6%) | 13 (12.9%) |  |
| 125,000-149,999 | 61 (6.4%) | 55 (6.5%) | 6 (5.9%) |  |
| ≥150,000 | 111 (11.6%) | 106 (12.4%) | 5 (5.0%) |  |
| Insurance |  |  |  |  |
| None | 41 (4.3%) | 35 (4.1%) | 6 (5.9%) | 0.0002 |
| Employer-sponsored coverage | 550 (57.7%) | 505 (59.2%) | 45 (44.6%) |  |
| Medicaid or state assistance | 93 (9.8%) | 72 (8.4%) | 21 (20.8%) |  |
| Medicare | 160 (16.8%) | 149 (17.5%) | 11 (10.9%) |  |
| Policy purchased on state/federal health exchange | 37 (3.9%) | 33 (3.9%) | 4 (4.0%) |  |
| Policy purchased on the commercial market | 29 (3.0%) | 25 (2.9%) | 4 (4.0%) |  |
| Tricare or VA benefit | 22 (2.3%) | 17 (2.0%) | 5 (5.0%) |  |
| Unsure | 22 (2.3%) | 17 (2.0%) | 5 (5.0%) |  |
| Geographical setting |  |  |  |  |
| Urban | 229 (23.8%) | 187 (21.8%) | 42 (41.2%) | <0.0001 |
| Suburban | 544 (56.6%) | 494 (57.5%) | 50 (49.0%) |  |
| Rural | 188 (19.6%) | 178 (20.7%) | 10 (9.8%) |  |
| Region |  |  |  |  |
| New England | 65 (6.8%) | 61 (7.1%) | 4 (3.9%) | <0.0001 |
| Mid-Atlantic | 128 (13.3%) | 106 (12.3%) | 22 (21.6%) |  |
| East North Central | 145 (15.1%) | 131 (15.2%) | 14 (13.7%) |  |
| West North Central | 53 (5.5%) | 48 (5.6%) | 5 (4.9%) |  |
| South Atlantic | 185 (19.2%) | 156 (18.1%) | 29 (28.4%) |  |
| East South Central | 61 (6.3%) | 48 (5.6%) | 13 (12.8%) |  |
| West South Central | 90 (9.4%) | 82 (9.5%) | 8 (7.8%) |  |
| Mountain | 74 (7.7%) | 74 (8.6%) | 0 (0.0%) |  |
| Pacific | 162 (16.8%) | 155 (18%) | 7 (6.9%) |  |
| Current AD severity |  |  |  |  |
| Clear | 29 (2.6%) | 22 (2.6%) | 1 (1.0%) | 0.15 |
| Mild | 238 (21.3%) | 191 (22.2%) | 14 (13.7%) |  |
| Moderate | 531 (47.5%) | 406 (47.2%) | 51 (50.0%) |  |
| Severe | 296 (26.5%) | 225 (26.1) | 32 (31.4%) |  |
| Current AD control |  |  |  |  |
| Minimally controlled | 259 (23.2%) | 199 (23.1%) | 23 (22.6%) | 0.02 |
| Somewhat controlled | 448 (40.1%) | 346 (27.2%) | 42 (41.2%) |  |
| Moderately well controlled | 300 (26.8%) | 234 (40.2%) | 25 (24.5%) |  |
| Very well controlled | 102 (9.1%) | 77 (8.9%) | 8 (7.8%) |  |
| Number of flare days in past 30 days |  |  |  |  |
| 0 | 44 (4.0%) | 35 (4.1%) | 2 (2.0%) | 0.18 |
| 1-3 | 271 (24.4%) | 205 (23.8%) | 28 (27.5%) |  |
| 4-7 | 206 (18.6%) | 158 (18.4%) | 27 (26.5%) |  |
| 8-10 | 151 (13.6%) | 123 (14.3%) | 11 (10.8%) |  |
| ≥11 | 437 (39.4%) | 339 (39.4%) | 34 (33.3%) |  |
| Comorbidities |  |  |  |  |
| Asthma | 382 (34.5%) | 300 (35.0%) | 34 (33.3%) | 0.74 |
| Allergic rhinitis | 557 (50.4%) | 435 (50.7%) | 49 (48.0%) | 0.61 |
| Food allergy | 426 (38.5%) | 322 (37.5%) | 46 (45.1%) | 0.14 |
| Frequent/persistent skin infections | 210 (19.0%) | 155 (18.1%) | 29 (28.4%) | 0.01 |
| Anxiety and/or depression | 404 (36.5%) | 326 (38.0%) | 25 (24.5%) | 0.008 |
| HCP visits in past year |  |  |  |  |
| 0 | 113 (10.6%) | 81 (9.4%) | 10 (9.8%) | 0.41 |
| 1-2 | 435 (40.6%) | 360 (41.9%) | 36 (35.3%) |  |
| 3-4 | 284 (26.5%) | 228 (26.5%) | 24 (23.5%) |  |
| ≥5 | 239 (22.4%) | 190 (22.1%) | 32 (31.3%) |  |
